# Dr. Charles A. Ring

**Published:** 1908-04

**Authors:** 


### OBITUARY

Dr. Charles A. Ring, formerly of Buffalo, died suddenly at Appleton, N. Y., February 25 1908, of heart disease aged 53 years. He was born at Buffalo, September 20, 1854, and received his academic education in the schools of his native city and at West Town School near Philadelphia. On his return to Buffalo, he became a student of medicine at the County House and graduated from the University of Buffalo in 1878. He served as resident physician at the County House, and in 1881 he was appointed medical superintendent of the insane, which position he held until April, 1889, when the insane were transferred to the state hospital at Collins. He then practised medicine in association with his brother, Dr. William G. Ring, at 364 Niagara Street in this city until 1892, when he was appointed by Surgeon General Walter Wyman to take charge of quarantine at Niagara Falls during the World's Fair at Chicago. At the close of this service Dr. Ring removed to Johnson's Creek, N. Y., where he practised his profession until early in 1894, when he returned to Buffalo. He married Miss Hannah D. Farrell, the daughter of Rev. Allen P. Ripley, a prominent clergyman of this city, in 1894, and the following year purchased Appleton Hall, at Appleton, for a summer home spending the winters in Buffalo.

Dr. Ring retired from active practice a few years ago. His wife died January 16, 1907. He is survived by a brother, Dr. William G. Ring, of this city. His father, the late Dr. William Ring, of this city, was a prominent practitioner of medicine for fifty years.

